# Functional thalamocortical innervation of VIP- and SST-expressing GABAergic interneurons in mouse barrel cortex

**DOI:** 10.1016/j.isci.2025.112539

**Published:** 2025-04-28

**Authors:** Michael Feyerabend, Mirko Witte, Martin Möck, Jochen F. Staiger

**Affiliations:** 1 Georg-August-University Göttingen, University Medical Center, Institute for Neuroanatomy, Kreuzbergring 36, 37075 Göttingen, Germany; 2 Department of Physiology and Pharmacology, University of Western Ontario, London, ON, Canada; 3 Robarts Research Institute, University of Western Ontario, London, ON, Canada

**Keywords:** Molecular biology, Neuroscience

## Abstract

In sensory cortices, conscious perception of stimuli involves a complex interplay of excitatory and inhibitory circuits. The subcortical source of sensory information is the thalamus, which in the whisker system houses (1) the “lemniscal” ventral posteromedial nucleus (VPM) and (2) the “paralemniscal” medial part of the posterior nucleus (POm). How much these two complementary inputs can drive feedforward inhibition in cortical layers by synapsing on GABAergic neurons is still incompletely understood. Here, we used optogenetics to study the incidence of VPM and POm projections to vasoactive intestinal polypeptide (VIP)- and somatostatin (SST)-expressing interneurons in barrel cortex. By using sensitive stimulation and recording methods, we show that VIP and SST neurons receive an extensive input, largely independent of laminar location or various cellular properties. Thus, thalamus-mediated feedforward inhibition is implemented in the circuit motifs of probably all VIP and SST interneurons, which they might differentially use, dependent on specific whisking behaviors.

## Highlights

• Lemniscal and paralemniscal input depolarizes most VIP and SST interneurons in barrel cortex
• These inputs show no obvious layer or cell type dependence
• Thalamus driven feedforward inhibition is a prominent circuit motif in barrel cortex function

## Graphical abstract

**Figure:**
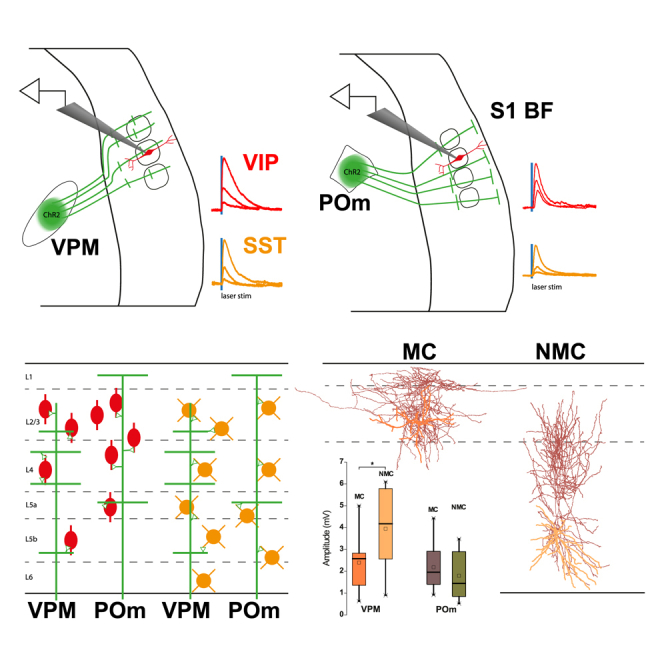

## Introduction

GABAergic interneurons are thought to play a key role in sensory neocortical processing due to their striking diversity.^1^^,^^2^ The functional significance of this repertoire of distinct subpopulations or even types of interneurons is ever evolving, yet, despite decades of progress,^3^ still not well understood.^4^^,^^5^^,^^6^ Many kinds of inhibition, each tied to a different subpopulation, have been proposed. Parvalbumin (PV)-expressing cells, for example, are thought to be specialized in mediating fast feedforward and feedback inhibition to narrow the impact of excitation to a time window of a few milliseconds^7^^,^^8^ and in agreement with this function receive the most robust thalamic input.^9^ As for the neurons under scrutiny here, somatostatin (SST)-expressing cells are considered to sculpt pyramidal cell ensembles due to their facilitating inputs to these principal cells by means of lateral inhibition,^10^ whereas vasoactive intestinal polypeptide (VIP)-expressing interneurons are mostly implicated in disinhibition.^5^^,^^11^ However, previous papers using optogenetic-assisted circuit mapping have shown weak, if any, innervation of SST interneurons by ventral posteromedial nucleus (VPM) and medial part of the posterior nucleus (POm) axons^9^ and by POm.^12^

Afferents from the VPM are the most prominent source of long-range excitatory input to the mouse barrel cortex (S1). They are also known as part of the lemniscal pathway, which is the primary relay of sensory information from the periphery.^13^^,^^14^ Apart from excitatory cells,^15^ its neocortical terminations strongly innervate PV cells in different layers,^9^ making them most responsive to whisker stimulation *in vivo*.^16^^,^^17^^,^^18^

However, much less is known on the innervation of GABAergic interneurons by the second major source of thalamic projections, the POm.^19^ Unlike VPM, it receives top-down input from various cortical areas (like S1^20^ and M1^21^) and its activity has not as strongly been associated to whisking compared to other active states. Hence, it has been proposed to convey already integrated sensorimotor signals. Its terminations, on the other hand, show a complementary innervation pattern to the lemniscal pathway, which could be suggestive of POm afferents being a second parallel stream of sensory information with a different quality^22^ but with a behavioral relevance for tactile perception.^23^

There is some evidence that other types of neocortical GABAergic neurons than PV cells receive direct input by VPM and POm. Tracing studies of thalamocortical afferents (TCAs) previously showed direct input to SST and VIP cells.^24^^,^^25^ Furthermore, some studies in acute brain slices suggest that non-PV interneurons receive functional direct input capable of the driving postsynaptic action potentials.^9^^,^^12^^,^^26^^,^^27^ The extent and strength of the TCA-to-nonPV interneuron circuit motif has recently come under further scrutiny by the use of channelrhodopsin-assisted circuit mapping (CRACM;^28^) with partly conflicting results, in particular in regard of the SST subpopulation (see, for example Sermet B.S.et al.^9^ and Audette N.J. et al.^12^). This issue is further complicated by SST cell diversity.^29^^,^^30^^,^^31^ VIP cells, on the other hand, are thought to be top-down integrators of various intracortical and neuromodulatory input.^32^^,^^33^^,^^34^ However, recently, they were also found to be involved in sensory representations.^18^^,^^35^ Consequently, their innervation by TCAs needs to be precisely characterized. Noteworthy in this context is the work by Williams and Holtmaat,^36^ which identified VIP cell activity driven by POm input as important gate of synaptic plasticity. Furthermore, VIP cells show a considerable amount of in-group diversity.^37^^,^^38^^,^^39^ It is unclear, however, how this diversity relates to innervation by TCAs.

Hence, this study used CRACM with a modified approach to probe both major thalamic afferents to S1 (VPM and POm) to VIP and SST cells through all layers. The crucial difference here is that optical stimulation was applied with a wide spectrum of different intensities for the benefit of detecting weaker connectivity/synapses. This way we could show that both, the lemniscal and paralemniscal pathways, comprehensively innervate cells belonging to 2 major subpopulations of GABAergic interneurons. Thus, they are also capable to implement feedforward dis-/inhibitory circuit motifs in the mouse barrel cortex that are considered to be integral parts of sensory processing and representation.^6^

## Results

### Experimental setting

**Figure 1:**
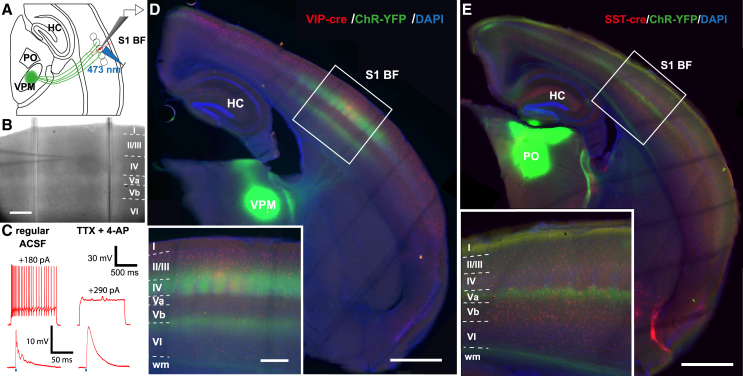
Thalamocortical injections and optogenetic stimulation (A) Schematic illustrating the experimental approach: While recording from a red fluorescent putative postsynaptic cell (here: a VIP cell), ChR2-YFP expressing fibers are stimulated locally with blue laser light. (B) Infrared image of the barrel field of an acute slice. The pipette indicates the location of the recorded cell, which is upper LIV. Scale bar: 300 μm. (C) After characterization of intrinsic properties, TTX and 4-AP are washed in to abolish AP generation. Left traces show a spike train during current injection and optically evoked postsynaptic potentials before the wash in. After the bath application of aforementioned drugs (right traces), current injection does not lead to spiking and postsynaptic responses became mono-component. (D) Example of a fixed slice with a VPM injection after completion of experiments. Insert shows endogenous fluorescence of ChR2-YFP and tdTomato driven by VIP-cre in the barrel cortex (S1 BF). Injection into VPM shows the typical lemniscal termination pattern with patchy signal in LIV and a band between LVb and LVI. (E) Example of a fixed slice with a POm injection after completion of experiments. Insert shows endogenous fluorescence of ChR2-YFP and tdTomato driven by SST-cre in the barrel cortex (S1 BF). Injection into POm shows the typical paralemniscal termination pattern with largely continuous bands in LI and LVa. *Roman numerals* depict cortical layers, *wm* – white matter. Scale bars of overviews: 1 mm, of inserts: 250 μm.

Optogenetic mapping of neuronal circuitry demands a specific transduction of the afferent fibers, i.e., in this study the respective thalamic nucleus. Representative examples of injections are shown in Figure 1 for VPM and POm. The profile of YFP-ChR2 expressing fibers within barrel cortex was used to control for specificity of the injection. The distinct patterns of cortical innervation were evaluated via vertical yellow fluorescent protein (YFP) expression profiling (Figure S1). Deviations from the expected pattern (peaks in LIV and at the LVb/VI border for VPM injections and peaks in LI and LVa for POm injections) led to exclusion of the slice for further analysis. Retrograde uptake by axonal projections of cortical cells, which would lead to YFP-expressing cells in LVI of barrel cortex, was never observed.

Genetically tagged cells of interest that were located within the terminal field of the thalamic projections were recorded in whole-cell current clamp mode (Figure 1). Beyond showing td-Tomato expression, the identity of recorded neurons was confirmed by intrinsic electrophysiological properties and post-hoc morphology for VIP cells (Figures S2 and S3) and SST cells (Figures S4 and S5). By washing in tetrodotoxin (TTX) and 4-AP after cell type characterization, we ensured that optical stimulation led to direct monosynaptic EPSPs (also known as CRACM^28^).

### Incidence and layer-specific distribution of thalamic inputs to VIP and SST neurons

**Figure 2:**
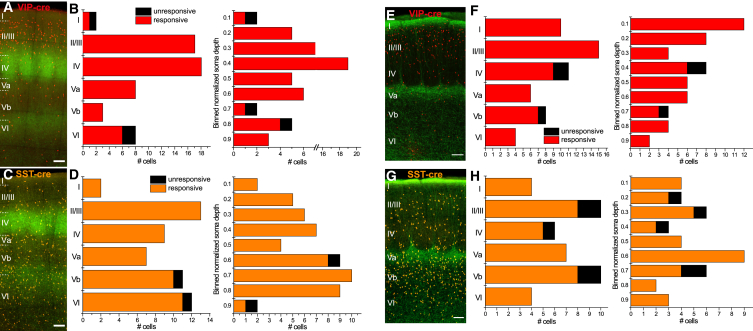
Incidence of optically induced thalamic input to VIP and SST cells in barrel cortex (A–C) (A) and (C) Maximum intensity projections of transduced fibers revealing lemniscal projection resulting from VPM injections. Cre-dependent red fluorescence illustrates the distribution of VIP and SST (pseudocolored orange) cell populations, respectively; scale bar: 100 μm. (B) Charts of responsive and unresponsive VIP cells according to soma location. The first bar plot shows the distribution according to layer and the second one according to normalized soma depth. 53 out of 56 cells were responsive to optical stimulation under TTX and 4-AP and were found throughout layers I to VI. (D) Charts of responsive and unresponsive SST cells illustrated in the same manner as in (B). 52 out of 54 cells showed responses. Cells without VPM input were found only in infragranular layers. (E–G) (E) and (G) Maximum intensity projections of transduced fibers revealing paralemniscal projection resulting from POm injections; scale bar: 100 μm. (F) Only 3 (out of 54) VIP cells, located in layers IV and Vb, were unresponsive to optical stimulation. (H) Distribution of responsive SST cells illustrated in the same manner. Here, 5 out of 41 cells (12.2%) were unresponsive, more broadly distributed across layers.

**Figure 3:**
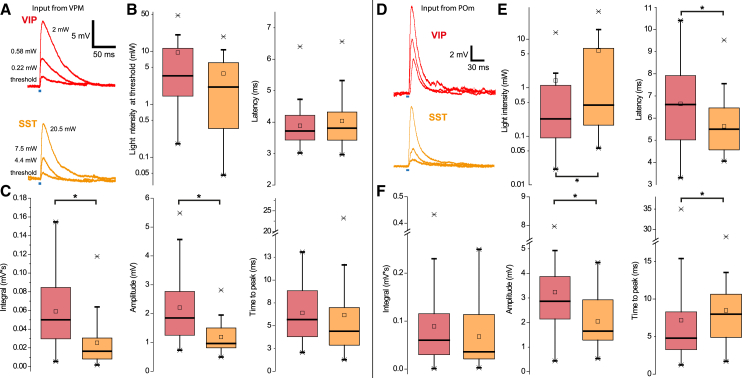
Comparison of thalamocortical synaptic properties in VIP versus SST cells (A–C) Input from VPM; (D–F) Input from POm. (A) Examples of optically evoked responses with increasing laser stimulation shown for a VIP and an SST cell. The blue marks delineate the 1 ms-long stimulus (duration not to scale). Note that responses can be overwhelmingly characterized by mono-component function suggesting singular synaptic events. (B) Stimulation intensity and EPSP latency between SST and VIP cells are similar. The little square within the boxplot indicates the average. (C) Inputs from VPM to VIP cells are significantly stronger in both EPSP integral (medians: 0.050 vs. 0.164 mV∗s, U = 110) and amplitude (medians 1.849 vs. 0.963 mV, U = 119); in both cases *p* < 0.001 (rank-sum test). Statistical significance of 0.05 or smaller is indicated by a singular asterisk throughout the figure. n_SST_ = 23, n_VIP_ = 24. (D) Examples of optically evoked responses from POm with increasing laser stimulation shown for a VIP and an SST cell. (E) Laser intensity necessary to evoke responses in SST cells is significantly higher (median: 0.23 vs. 0.44 mW; rank-sum test, U = 255.5, *p* = 0.011), whereas latencies in VIP cells are higher and more variable (median: 6.6 vs. 5.5 ms; rank-sum test, U = 289, *p* = 0.030). (F) Overall input strength quantified by EPSP integral is not significantly different, rank-sum test, U = 371, *p* = 0.353). However, VIP cells have responses with higher amplitudes (median: 2.87 vs. 1.65 mV; rank-sum test, U = 235, *p* = 0.003), whereas SST cells have a longer rise time (median: 4.8 vs. 8.0 ms; rank-sum test, U = 271, *p* = 0.014). n_SST_ = 27, n_VIP_ = 32.

Generally, no layer was devoid of input for either thalamic nucleus or cell type (Figure 2). By using a careful experimental approach, an overwhelming majority of VIP and SST cells showed postsynaptic responses, given sufficient stimulation (Figures 3A and 3D). In VPM injections, 53 out of 56 VIP cells (94.6%) were responsive to optical stimulation. The unresponsive cells were located at the poles of the cortical column. LI showed the highest incidence of cells lacking direct innervation. However, results should be interpreted with caution due to small sample size (*n* = 2) that is attributable to our strategy to sample a number of cells per layer that is reflecting their overall occurrence there. SST cells showed a similarly frequent innervation with 52 out of 54 (96.3%) responding cells. The two unresponsive cells were located in LVb and LVI. When pooling both layers, 4 out of 5 cells showing no innervation are located in infragranular layers. However, an observer-independent analysis according to distance to the pial surface does not show that these cells cluster at a particular depth within a certain layer (or at a laminar boundary; see Figures 2B and 2C).

Direct stimulation of POm terminals showed a similar pattern of abundant innervation. Responsive cells could be found in all layers for both cell types. Unresponsive cells could be found in layers with the sparsest projections, i.e., LIV, LVb, and LVI and were a little more frequent in SST than in the VIP subpopulation (12.2% vs. 5.6%). In order to test which factors (i.e., cell type, layer location, and used intracellular solution) were more suitable at predicting input or lack thereof from the respective thalamic nucleus, we performed stepwise logistic regressions with multinominal predictors for each POm and VPM. The model based on VPM incidence, was significant (Chi^2^ vs. constant: 25.5, *p* < 0.001, McFadden’s R^2^ 0.549) using intracellular solution and layer location but interestingly not cell type as predictors. Addition of predictors in the POm model did not provide a better fit than a constant.

### Properties of optically induced EPSPs and their relationship to VIP and SST cells

In order to account for variability in expression levels of putative presynaptic fibers, optically evoked EPSPs were compared at threshold stimulation levels. The interquartile range (IQR) of laser intensities needed to evoke reliable responses varies about an order of magnitude (Figure 3B). Optically evoked responses were predominantly monocomponent, suggesting either release from one single source or multiple highly synchronous ones. Synaptic latencies ranged from 2.97 to 6.56 ms and decreased with higher stimulus intensity. Distribution of latencies was similar between VIP and SST cells with an overall median of 3.73 ms (23 SST vs. 24 VIP cells). In terms of input strength there was a considerable difference: responses in VIP cells had a much higher EPSP integral (medians: 0.164 vs. 0.050 mV∗s; P 0.001) and amplitude (medians 1.849 vs. 0.963 mV; P 0.001) than their SST counterparts. Of note, time to peak, which is also influenced by overall size of the response, was not significantly different. Given the stark difference in EPSP integral between the VIP and SST cell populations, the expectation would be that SST cells have significantly lower rise times. Since this was not the case, evoked responses in SST cells are not scaled-down versions of those in VIP cells but also show a difference in kinetic properties with less steep rising phases.

**Figure 4:**
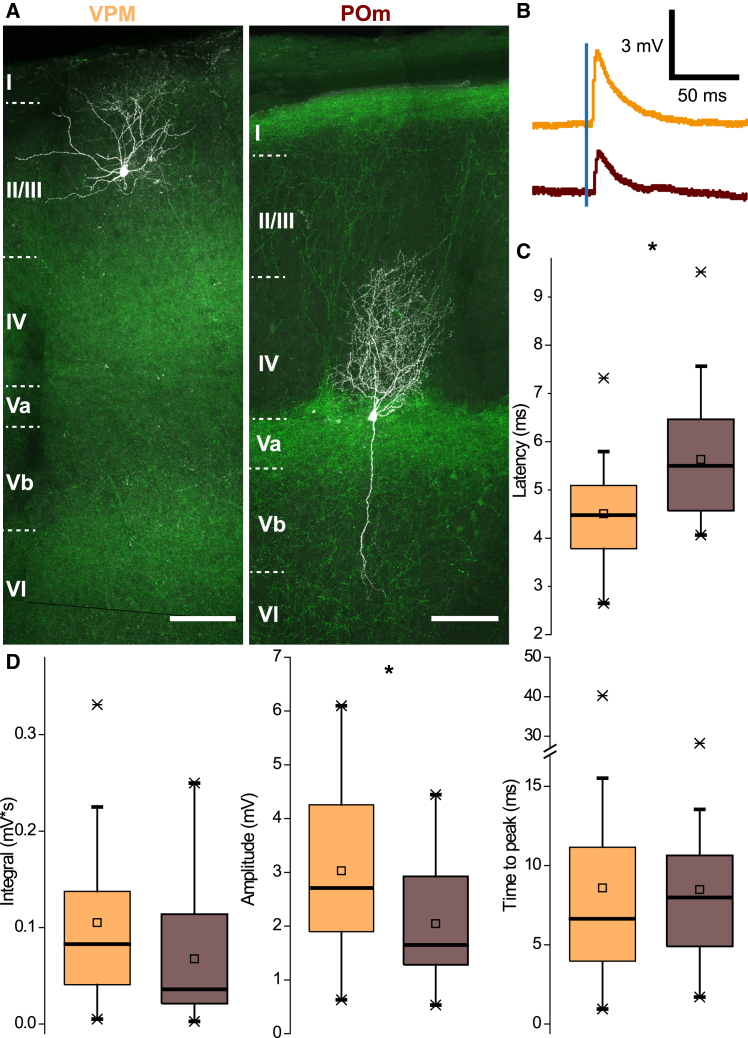
Comparison of synaptic properties in SST cells according to thalamic source nucleus (A) Examples of two biocytin-filled SST cells with transduced fibers of VPM and POm. Scale bar: 100 μm. (B) Example of threshold responses according to the respective fiber populations; VPM in orange, POm in brown. (C) Evoked responses by POm stimulation have a longer latency (median: 4.5 vs. 5.5 ms, rank-sum test,*p* = 0.001, U = 147). Statistical significance of 0.05 or smaller is indicated by a singular asterisk throughout the figure. (D) Integrals of EPSPs upon POm stimulation have a tendency to be smaller (median: 0.0828 vs. 0.0360; *p* = 0.095), whereas peak amplitudes are clearly lower (mean: 3.03 vs. 2.05 mV; t test, *p* = 0.018, t(49) = 2.441). Analysis based on 24 cells for VPM and 27 cells for POm.

Unlike in Figures 3A–3C, where K^+^-based intracellular solution was used, subsequent analysis refers to responses recorded with Cs^+^-based solution (27 SST vs. 32 VIP cells). Hence, parameters described there cannot be directly compared with the previous section. Under electrotonic more compact conditions, higher stimulation intensity of POm fibers was needed to evoke threshold responses in SST cells (median: 0.23 vs. 0.44 mW; *p* = 0.011). VIP cells had considerably longer latencies (median: 6.6 vs. 5.5 ms; *p* = 0.030). In addition, they showed a much higher variability in response times (range: 7.1 vs. 5.46 ms; IQR: 3.05 vs. 1.89 ms). Light-induced EPSPs of SST cells had considerably smaller amplitudes (median: 2.87 vs. 1.65 mV; *p* = 0.003), whereas overall input strength quantified by their integral was comparable (*p* = 0.357). Consequentially, SST cells showed longer rise times (median: 4.8 vs. 8.0 ms; *p* = 0.015), which makes up for smaller amplitudes. A sample of SST cells recorded with Cs^+^-based solution was sufficient to compare the optical stimulation of fiber populations (Figure 4). Threshold responses upon POm stimulation had a significant longer latency (median: 4.5 vs. 5.5 ms, *p* = 0.002, Figure 4C). The integral of EPSPs evoked by VPM fibers was similar in VIP and SST cells (Figure 4D) (median: 0.0828 vs. 0.0360 mV∗s; *p* = 0.095). Amplitudes, on the other hand, were substantially higher upon VPM stimulation (mean: 3.03 vs. 2.05 mV; t test, *p* = 0.014), whereas rise times of EPSPs were comparable.

### Properties of optically induced EPSPs in SST cells and their relationship to thalamic source nucleus

Previous research on VIP cells did not show unambiguous subtypes (cf. Gouwens N.W. et al.^38^ and Prönneke A. et al.^39^). Thus, we refrained from a deeper analysis of input to VIP cells at this point. However, SST cells can be distinguished by morphology (Martinotti, MCs, versus non-Martinotti cells, nMCs) with corresponding electrophysiological features (quasi fast-spiking versus other action potential firing types^29^^,^^38^). Consequentially, the question arose if these SST subtypes show differences in synaptic responses upon optical stimulation of thalamic axons. In order to include both VPM and POm injections into the analysis, subtypes were identified by morphology and characterization of biophysical properties (as much as it was possible with a Cs^+^-based intracellular solution). Since SST subpopulations showed robust and distinguishable morphological traits (Figures 4A, S4, and S5), it was possible to associate them with basic subthreshold parameters. Cells, identified as either MCs or nMCs, displayed strong differences in R_in_ and membrane time constant. These parameters were thus used to tentatively classify SST cells, which could not be morphologically identified due to incompleteness of the axonal tree or insufficient staining.

**Figure 5:**
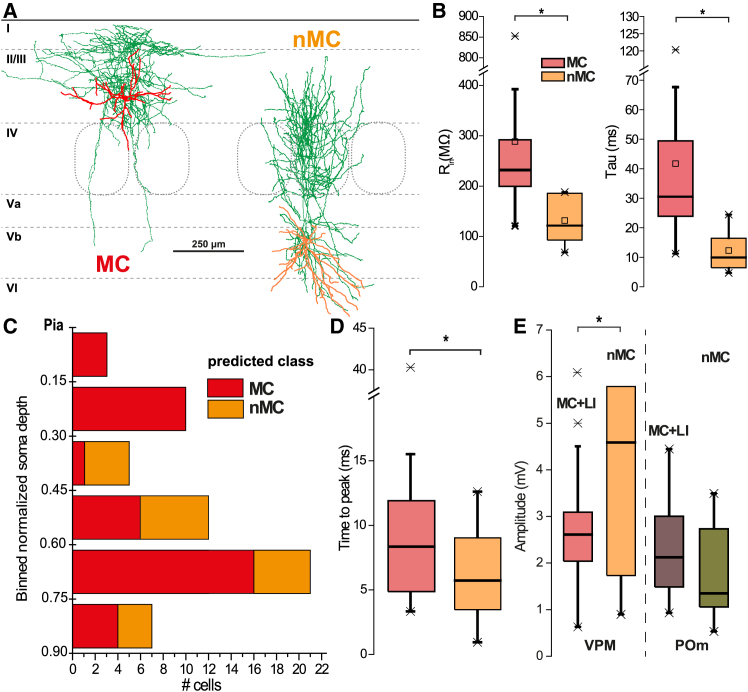
Comparison of thalamus-evoked synaptic properties in SST cells according to morphological type (A) Examples of reconstructions of an MC (characterized by an axonal plexus in LI as shown on the left) and a non-MC (with the absence of axonal branching in LI). (B) Recordings with Cs+-based IC solutions showed differences between morphologically identified cells (16 MC and 7 non-MC) in input resistance (medians: 232.0 MOhm vs. 121.5 MOhm, rank-sum test: *p* ≤ 0.001, U = 243) and time constant (medians: 30.51 ms vs. 9.94 ms, rank-sum test: *p* = 0.002, U = 243). Statistical significance of 0.05 or smaller is indicated by a singular asterisk throughout the figure. (C) Distribution of SST subtypes across normalized soma depth. (D) Responses of non-MCs have a significantly shorter rise time. Data are pooled from the two nuclei, since type of thalamic projection was not a significant factor (*p* = 0.4327, F(1, 46) = 0.63) in a two-way ANOVA with SST subtype as other factor (*p* = 0.0356, F(1, 46) = 4.69). There was no interaction effect: *p* = 0.1948, F(1, 46) = 1.7. (E) Amplitude of responses showed a significant interaction effect (*p* = 0.0425, F(1, 46) = 4.35) between cell type (*p* = 0.4901, F(1, 46) = 0.48) and injected thalamic nucleus (*p* = 0.0029, F(1, 46) = 9.87) due to VPM stimulation of non-MCs in a two-way ANOVA; *n* = 50 (24 VPM vs. 26 POm, pMC = 34, pNMC = 16).

We used a K-nearest-neighbor classifier (see STAR Methods for details), trained with 23 morphologically identified SST cells and a validation performance of 82.6%, to predict other SST cells recorded with Cs-based solution. Classification of these cells yielded a distinct laminar distribution with nMCs being concentrated in granular and MCs being concentrated in supragranular and infragranular layers (see Figure 5C). We then performed a two-way ANOVA on response properties of non-L1 SST cells (*n* = 58) recoded with cesium-based solution with morphological type and thalamic pathway as factors. Subtype had a significant effect but not thalamic nucleus (*p* = 0.036 vs. *p* = 0.433) or rise time (see Figure 5D for distribution by subtype) In addition, type of stimulated fiber and cell identity showed a significant interaction effect onto response amplitudes (Figure 5E, two-way ANOVA, *p* = 0.043): responses of nMCs, when stimulating VPM projections, were higher than of MCs (medians: 4.60 vs. 2.62mV), whereas they were comparable in the POm stimulation condition. This finding suggests that lemniscal innervation of nMCs is by far the most robust version of thalamic innervation of SST cells.

## Discussion

The main result of this study is that both VIP and SST cells are frequently and directly innervated by VPM and POm, which enables them to participate in sensory stimulus-driven feedforward inhibition of barrel cortex.^6^^,^^18^ The approach of using a variable stimulation strength reveals that SST cells are almost as commonly innervated as VIP cells, albeit at presumably lower strength. This finding appears to be odds with some previous studies that report that SST cells are devoid of innervation by paralemniscal fibers, even when tested under the same pharmacological treatment^12^^,^^36^ but there are technical details that may offer an explanation.

### Technical considerations

Optogenetics is a powerful method to specifically stimulate ChR2-transduced neurons of interest.^32^^,^^40^ Due to thalamic inputs being powerful and capable of driving innervated target cells to fire, in principle, disynaptic excitation of the GABAergic neurons recorded from, originating from intracortical sources, is conceivable.^26^ However, by applying the CRACM variety of optogenetic stimulation,^28^ we made sure that this could not happen. Therefore, our excitatory inputs on VIP or SST neurons in barrel cortex have to originate from the two thalamic nuclei under study here.

Of note here is that POm innervation of barrel cortex is less dense than VPM innervation and that its excitatory input to SST cells appears to lead to rather meek postsynaptic responses (at least under single pulse stimulation). In addition, using CRACM pharmacology^28^ requires a stronger stimulation regimen and does not follow the binary logic of spike/no-spike anymore. Hence, it seems very plausible that using this approach is disadvantageous for sufficient recruitment of POm-to-SST-cell synapses, which might explain some of the discrepancy with previous studies. Here, we carefully “titrated” the laser power to achieve an optimal stimulation intensity. In addition, we used an intracellular Cs^+^-based solution and recorded at −70 mV holding potential to increase the driving force for the incoming EPSPs. When comparing response properties between both nuclei (see Figure 4D), POm input to SST cells had a lower amplitude, longer rise time and latency than VPM input. These observations are in line with paralemniscal input being more distal in its subcellular targeting. Furthermore, other more recent studies show that SST cells can be innervated by long-range inputs (i.e., POm) particular in layers with high-density terminations.^9^^,^^41^

### Thalamic innervation of VIP cells

The data presented in this work firmly establish the functional thalamocortical innervation of VIP cells by the two main afferent nuclei VPM and POm, as previously suggested by Rabies tracing.^25^^,^^42^ There is some evidence that VIP cells are particularly susceptible to sensory experience-dependent plasticity, in comparison to other GABAergic interneurons.^43^ Overall, recent research has placed them as conveyer of top-down input from other cortical areas and neuromodulatory sources.^32^^,^^33^^,^^44^^,^^45^^,^^46^ However, recent evidence also suggests core sensory representation features being integrated in the activity of VIP neurons.^18^^,^^35^ Hence, also the direct influence of immediate sensory information from the periphery must be considered in understanding VIP function. This more general capacity to be driven by various sources outside the local network makes them central hubs of brain-wide information processing. The question of how these diverse inputs are translated into VIP cell activity arises, particularly given their wide-ranging vertical extent of dendritic arborizations as seen in pyramidal cells.

### Thalamic innervation of SST cells

There are some notable differences in the functional properties of optical fiber stimulation effect in VIP versus SST cells: EPSC amplitude at threshold stimulation is higher in VIP cells for both VPM and POm input. Moreover, overall synaptic input strength of the lemniscal pathway—quantified by the EPSP integral—is substantially stronger in VIP cells. These differences substantiate that SST inputs are relatively weak. However, their slower rise time give them a longer time window for temporal summation. Furthermore, it is reasonable to assume that thalamocortical synapses onto SST cells are facilitating as it is known for intracortical excitatory and inhibitory input.^47^^,^^48^^,^^49^ Hence, given sustained downstream activity, SST cells could be a possible mediator of feedforward inhibition in the upper dynamic range of incoming activity.^27^^,^^50^

Other non-PV interneurons, not targeted in this study, are also known to receive thalamic input.^41^^,^^51^ In short, a neuron population that does not have the potential to receive TCAs in barrel cortex is hard to come by. Ultimately, the question arises if TCAs have a cell type specific quality at all? To the best of our knowledge, the only report of a specific cell type being in violation of Peter’s rule in regard of long-range connectivity is by Petreanu et al.,^40^ in which L5b cells do not show POm input despite a considerable overlap between thalamic axons and target cell dendrites. The authors of the study speculate that these cells themselves are projecting to the POm and a direct disynaptic loop between the areas appears to be avoided. Hence, excitatory projection cells in infragranular layers (also home of most SST cells) show potentially a much higher variability in TCA input than GABAergic interneurons.

In conclusion, the advent of optogenetics has led to many new exciting opportunities for cell type targeted investigations of cortical microcircuitry. However, studies using singular pulses that are designed to infer incidence of connectivity ought to make sure that weaker synapses are stimulated sufficiently. Quantitative conclusions on TCA input strength should be done cautiously. In fact, synaptic vesicle release from thalamocortical fibers *in vivo* can occur under a wide range of conditions (i.e., short-term dynamic, behavioral state, neuromodulation, etc.) and presumably engages different cell types in different contexts. More research on how sensory activity directly drives different GABAergic interneurons *in vivo* is needed before determinations, such as SST cells receive weak and VIP cells negligible TCA-mediated drive, can be made.^17^ An interesting approach in this context is taken by Gasselin et al.,^34^ who showed that VIP cell activity associated with sensory stimulation is stronger during active whisking due to additional and selective excitation by the cholinergic system. Last but not least, rare infragranular cell type specific recordings show that some SST cells are engaged in active sensory processing whereas others are inhibited.^52^ In the same direction, recent work by Guy et al.^18^ presented evidence that passive whisker stimuli also do largely inhibit layer II/III SST cells but some do show weak depolarizing input, sometimes in a whisker stimulus direction-dependent manner. Future research should elucidate by which circuit mechanisms these differences come about.

### Limitations of the study

Our study sought to determine basic functional connectivity patterns of feedforward inhibition in cortical circuits. Since a study of this kind relies on acute slice preparations, slice artifacts, i.e., the truncation of neurites (dendrites as well as axons), are always existent. As such they could lead to false-negative findings. To minimize this confounder, we used CRACM-based optogenetics where we stimulated the axon terminals in the cortical area of interest. Therefore, a specific stereotactic injection of a thalamic nucleus should lead to a comprehensive mapping of these fibers’ inputs to target cells of interest, in this case VIP and SST-expressing interneurons.

## Resource availability

### Lead contact

Further information and requests for resources and reagents should be directed to and will be fulfilled by the lead contact, Jochen F. Staiger (jochen.staiger@med.uni-goettingen.de).

### Materials availability

This study did not generate new unique reagents. Further information and requests for resources and reagents should be directed to and will be fulfilled by the lead contact.

### Data and code availability

• The published article and supplemental information include all data generated and analyzed during this study.
• The original code written in Signal 5 for analysis of intrinsic properties and evoked postsynaptic responses can be obtained on Zenodo (https://doi.org/10.5281/zenodo.15013697). Analysis code written in MATLAB can be obtained there as well (https://doi.org/10.5281/zenodo.15208531).
• Any additional information required to reanalyze the data reported in this paper is available from the lead contact upon request.

## Acknowledgments

We thank Patricia Sprysch for expert technical support and Pavel Truschow for help with the imaging. This study was supported by a grant from the 10.13039/501100001659Deutsche Forschungsgemeinschaft (DFG STA 431/14-1).

## Author contributions

M.F., M.M., M.W., and J.F.S. conceived the experiments; M.F. collected the data; MF., M.M., and M.W. analyzed the data and interpreted the results; M.F and J.F.S. wrote the manuscript; all authors edited the manuscript and approved its final version.

## Declaration of interests

The authors declare no competing interests.

## STAR★Methods

### Key resources table

**Table:** 

REAGENT or RESOURCE	SOURCE	IDENTIFIER
**Antibodies**		

goat polyclonal antiGFP (original concentration 0.5 mg/ml)	abcam, UK	ab6673
donkey anti goat Alexa 488	Thermofisher, USA	A-11055

**Bacterial and virus strains**		

AAV5.hSyn.hChR2(H134R)-eYFP.WPRE.hGH, original titer: 8.62 × 10e12 GC/ml	UPenn Vector Core, USA	addgene plasmid id: 26973P
AAV9.hSyn.hChR2(H134R)-eYFP.WPRE.hGH, original titer: 1.42 × 10e13 GC/ml	UPenn Vector Core, USA	addgene plasmid id: 26973P

**Chemicals, peptides, and recombinant proteins**		

TTX	Tocris Bioscience, UK	Cat. No. 1078
4-AP	Sigma-Aldrich, USA	275875-1G
Streptavidin-Alexa633	life technologies, USA; now ThermoFisher	S32357
DAPI	life technologies, USA; now ThermoFisher	

**Experimental models: Organisms/strains**		

Mouse reporter strain: Gt(ROSA)26Sor^tm9(CAG-tdTomato)Hze^	The Jackson Laboratories, USA	Stock No. 007905
Mouse cre driver line 1: Vip^tm1(cre)Zjh^	The Jackson Laboratories, USA	Stock No. 010908
Mouse cre driver line 2: Sst^tm2.1(cre)Zjh^	The Jackson Laboratories, USA	Stock No. 028864

**Software and algorithms**		

Neurolucida	MBF Bioscience, USA	
arivis vison4d	Arivis, Germany	
SigmaPlot 13	Systat Software, USA	
MATLAB	MathWorks, USA	https://doi.org/10.5281/zenodo.15208531
Signal 5	CED, UK	https://doi.org/10.5281/zenodo.15013697

**Other**		

PowerMax Wand UV/VIS Quantum	Coherent, USA	
DL-473	Rapp OptoElectronic, Hamburg, Germany	

### Experimental model and study participant details

Animals of either sex were used as heterozygous offspring crossed by the respective homozygous Cre driver line and the homozygous reporter line (see key resources table). All mouse lines were originally acquired from The Jackson Laboratory (Bar Harbor, Maine, USA) and were bred to maintain a C57BL/6 genetic background. Animals were kept on a 12 hour light-dark cycle with *ad libitum* standard diet. All experiments were conducted in accordance with the German Animal Welfare Act and corresponding EU legislation. Permission was obtained by the Landesamt für Verbraucherschutz und Lebensmittelsicherheit Niedersachsen (LAVES) under the reference number 33.9-42402-04-15/1897. Experimentally naive animals were used for stereotactic injections, which was performed shortly after weaning between postnatal day 21-28.

### Method details

#### Virus injections and slice preparation

Before surgery mice were injected intraperitoneally (30 μL per 10 g body weight) with buprenorphine (0.3 mg/mL) diluted 1:14 with sterile saline for analgesia. Mice then were placed on a heating plate set to 37°C with the head mounted in a stereotactic frame. Throughout the surgery animals were subjected to a constant flow of pure oxygen gas mixed with isoflurane (1-3% isoflurane applied by adjustable vaporizer). An eye ointment was applied to prevent drying or irritation of the eyes.

After shaving of the head, the scalp was injected with lidocaine (10 mg/mL) for local anesthesia. Then, the rostro-caudal head angle was adjusted until lambda and bregma had the same z-coordinates. The cranial windows for both hemispheres were made at the respective coordinates with a dental drill with injection coordinates were taken from the Paxinos mouse atlas (see [Franklin and Paxinos, 2001]; VPM: 1.7, 1.75, -3.25; POm: 2.16, 1.25, -3.00, axis: anterior-posterior, medial-lateral, dorsal-ventral, all in mm) multiplied with a correction factor to account for juvenile animals. It was calculated by dividing the measured distance between bregma and lambda by 4.2 mm.

Due to uneven growth of the brain, the coordinates on the dorsal-ventral axis were determined by the following formular: -(2/3 (1-CF) + CF) ∗ Paxinos coordinate. Injection pipettes were made from glass capillaries with a P-97 horizontal puller in a two-step program to produce long and thin tapering. Tips were then trimmed under a microscope to have an opening of 20–25 μm in diameter. Virus was front-filled from droplets and approximately 100–150 nl of virus solution was delivered at the respective depth by pressure injection (parameters: 3 psi, 250 ms). After a settling period of several minutes, pipettes were removed, and the head was sutured. For post-operative care animals were injected two more times with buprenorphine at 4 and 8-10 hours after the surgery.

AAV-Virus was obtained from the Vector Core of the University of Pennsylvania (Philadelphia, USA). A detailed description of the used vectors and their original titer is given in key resources table. Upon arrival all obtained viruses were aliquoted on ice 1:4 in sterile PB, under a laminar flow hood into batches of 5–10 μl each and stored at −80°C. Aliquots were not used more often than 2-3 times to ensure that consecutive thaw-freeze cycles did not lower the titer drastically.

3 to 4 weeks after the injection, animals were used for in-vitro experiments with acute slices. Upon anesthesia with isoflurane, the brain was quickly removed and placed into iced high-sucrose preparation solution (NaCl 87 mM, NaH2PO4 1.25 mM, KCl 2.5 mM, glucose 10 mM, sucrose 75 mM, CaCl2 0.5 mM, MgCl2 7 mM, NaHCO3 26 mM). Both cutting and recording solutions were prepared fresh from 10X stocks, with NaHCO3 added separately, and constantly perfused with carbogen gas (95% CO2 5% O2). 300μm-thick slices containing the barrel field of the primary somatosensory cortex were collected and transferred to recording solution (NaCl 125 mM, NaH2PO4 1.25 mM, KCl 2.5 mM, glucose 25 mM, CaCl2 2 mM, MgCl2 1 mM, NaHCO3 26 mM). After cutting, slices were rested for 30 min at 37°C.

#### Intracellular recordings

Cells were recorded from with two different kinds of intracellular solutions: based on (i) K+-gluconate (K-Gluconate 135 mM, KCl 5 mM, EGTA 0.5 mM, HEPES 10 mM, Mg-ATP 4 mM, Na-GTP, 0.3 mM, Na-P-creatin 10 mM) or (ii) Cs+-MeSO4 (CsMeSO4 135 mM, CsCl 5 mM, EGTA 0.5 mM, HEPES 10 mM Mg-ATP 4 mM, Na-GTP 0.3 mM, Na-P-creatin 10 mM). Aliquots of 0.5 ml intracellular solution were made in bulk as follows: the pH of solutions was adjusted to 7.4 by adding either KOH or CsOH, while monitored with a pH-meter; then, the solution’s osmolarity was assessed with a Gonotec OSMOMAT 030 and adjusted to 300 mOsm by adding sucrose. Aliquots were sterile filtered and frozen at −20°C. Shortly before experiments, an aliquot was thawed and 0.5% biocytin was added for labeling of the recorded cell. Before filling of the patch pipette, solution was passed through an additional syringe filter (polytetrafluoroethylene, sterile, pore size 0.2 μm).

Recordings were made with a single electrode patch-clamp amplifier (SEC-05; npi electronics) connected to a low-noise headstage. To avoid series resistance errors, measurements were made in discontinuous mode with a switching frequency above 20 kHz and a 1/4 duty cycle. The amplifier included a low pass Bessel filter with the corner frequency set to 3 kHz. In addition, signals were also subjected to a filter module (DPA-2FX; npi electronics) with manual offset correction and a single pole lowpass filter with corner frequency of 20 kHz. Ultimately, recordings were digitized at 20 kHz by an AD-Converter (CED Power1401, CED Limited, Cambridge, England). Tissue and single cells were visualized with an upright microscope (Axio Examiner A1, Carl Zeiss Microscopy, Jena, Germany) using two different objectives: a 2.5x (EC Plan-Neofluar 2.5x, NA = 0.075, working distance = 9.5 mm, Carl Zeiss Microscopy) and a 40x water immersion objective (LUMPlanFl W/IR, NA = 0.80, working distance = 3.3 mm, Olympus Corporation, Tokyo, Japan). For brightfield microscopy with infrared light, a halogen lamp (housed in HBO 100 illuminator, Carl Zeiss Microscopy) was filtered and coupled into a Dodt gradient contrast system (Dodt et al., 1998). Epifluorescence was enabled with light of a mercury arc lamp (HXP 120 C, Carl Zeiss Microscopy) that was filtered by a dichroic mirror. Light of both optical paths was ultimately captured by a CCD camera (INFINITY3S-1UR, Lumenera corporations, Ottawa, Canada). Micromanipulators (SM5, Luigs & Neumann, Ratingen, Germany) were used for moving the headstage and recording chamber to control patch pipette and image plane. Most parts of the set-up were mounted on an IsoStation vibration isolated workstation (Newport Corporation, Irvine, USA) and placed within a Faraday cage. Constant perfusion with recording solution was achieved by a peristaltic pump (Minipuls 3, Gilson, Middleton, WI) at a rate of about 2 ml min−1. Before entering the recording chamber, ACSF was heated up at 34°C with a heating pen and maintained in the recording chamber with a temperature controller (TC05, Luigs and Neumann). To prevent noise, electrical components were individually connected to ground.

Patch pipettes were made shortly before experiments with a horizontal puller (P-1000, Shutter Instruments) using 4 cycles. When immersed into the recording solution pipettes had a resistance of 6–10 MOhm. After immersion, slight positive pressure was applied through the tip. Then, slow capacitance compensation was done manually, when the pipette was in proximity to the slice surface. The target cell was approached swiftly at a horizontal angle of about 45°. After dimpling of the membrane, the pressure was reversed and the amplifier switched to VC with a command voltage of −60 mV. Pipette tip and membrane then usually formed a tight seal of a resistance beyond 1GOhm. Renewed manual compensation of the capacitance was done according to 1 s long hyperpolarizing 100 pA current pulses. Stimulation was applied via a gating unit (GIA-05X; npi electronics). Acquisition protocols were custom-designed in Signal 5 (CED Limited). Measured values were not corrected for liquid junction potential. These were estimated to be +14 mV for Cs+ based intracellular solution and +16 mV K+-based solution. Optogenetic stimulation was applied with a solid-state laser (lambda = 473 nm, DL-473, Rapp OptoElectronic, Hamburg, Germany). The laser output was coupled into the microscope via a glass fiber light cable (200 μm, NA = 0.22) and a beam combination cube containing a dichroic mirror for the appropriate wavelength (all Rapp OptoElectronic).

After assessment of intrinsic properties, tetrodotoxin (TTX, 1 μM) and 4-aminopyridine (4-AP, 100 μM) were washed in by bath application. Intensity of laser stimulation was increased in a step-wise manner, with at least three repetitions at each level at an inter-stimulus interval of either 5 or 15 s. The different conditions did not influence variability of amplitude or latency of the responses, which would have hinted at different recruitment of ChR2 by desensitization and were consequently pooled. Intensity level started at subthreshold levels (approximately 10–30 μW) and were increased in a step wise manner, which could reach up to 50 mW. Intensity of transmitted light passing the objective was regularly measured in dry conditions with a photometer (PowerMax Wand UV/VIS Quantum, Coherent, Santa Clara, USA). The holding current applied to the cells was adjusted manually to a baseline membrane potential of -70 mV. The first postsynaptic events that reliably followed a 1 ms light flash within a consistent time window (latency range of 1.5 ms) were considered threshold stimulation of transduced fibers. If a cell was unresponsive, a positive control in the same or neighbouring slice was obtained. After in-vitro experiments, slices were fixed overnight in a solution of 4% PFA and picric acid at 4°C.

#### Histology and imaging

Slices were washed in PB (0.1 M, pH 7.4 : 14.42 g Na2HPO4 + 2.62 g NaH2PO4, dissolved in 1 l ultrapure water) several times until they were completely destained of the yellow picric acid. Then they were transferred into a saline solution by first washing them 15 min with TB buffer (0.05 M, pH 7.6: 6.06 g tris(hydroxymethyl)aminomethane hydrochloride +1.39 g tris(hydroxymethyl)aminomethane base, dissolved in 1 l ultrapure water), followed by washing in TBS (TB buffer + 0.9 m/v % NaCl) for 15 min. For permeabilization they were washed two times for 15 min each in TBS with additional 0.5% Triton-X 100 (TBST). In some POm injections the YFP signal was immunohistochemically amplified. In these cases, slices were incubated for 90 minutes in TBST with additional blocking agents, to limit unspecific binding of primary and secondary antibody. Then, slices were incubated for 2-3 days at 4°C–8°C in a solution of the same composition as in the previous step, but with the addition of goat polyclonal antiGFP (abcam, Cambridge, UK; original concentration 0.5 mg/mL diluted 1:4000). Further on, slices were washed for five times each 15 minutes in TBST. For staining of biotin and optional amplification with the secondary antibody, streptavidin conjugated to the fluorophore Alexa633 (life technologies, Carlsbad, USA) and donkey anti goat coupled to Alexa488 was added and incubated for 4 hours. After that, slices were washed again 3 times in TBST and transferred in TBS. Then, cell nuclei were stained with 4,6-diamidino-2-phenylindole (DAPI, life technologies; 5 mg/ml, diluted 1:1000) for 5 min, washed one time in TBS and two times in TB and ultimately mounted with aqua polymount (Polysciences, Warrington, USA) on specimen slides with 0.08–0.12 mm thick cover slips (Menzel, Thermo Scientific, Waltham, USA).

For evaluation of cellular morphology and the thalamocortical injection pattern, partial image stack acquisitions of the barrel field were made with a confocal microscope (TCS SP2, Leica Microsystems, Wetzlar, Germany) using a 40x oil-immersion objective. Single tiles of confocal image stacks were stitched either manually in arivis vison4d (arivis, Munich, Germany) or automatically with an ImageJ-plugin (Schneider et al., 2012). Single cells, which showed sufficient completeness of neuronal arborizations, were selected for reconstruction. Morphology was reconstructed manually in Neurolucida (MBF Bioscience, Williston, USA) from acquired image stacks. No shrinkage correction due to fixation of the tissue was applied.

### Quantification and statistical analysis

#### Intrinsic properties

Analysis of intrinsic properties was done by characterizations of 48 cells from 23 animals in case of VIP neurons and 40 cells from 15 animals in case of SST neurons. All electrophysiological measurements were made in CC and analyzed by custom-written scripts (Signal 5, CED). Subthreshold intrinsic properties (except rectification index and rheobase) were obtained from 10 iterations of a one second stimulation with -10 pA or, alternatively, -50 pA if necessary for a dependable response. The resting membrane potential Vm (in absolute voltage) is measured as the average pre-stimulus membrane voltage. The input resistance Rin is determined according to Ohm’s law by the potential change measured from rest to highest deflection. The time constant tau was extracted from the average of the same traces. It is calculated as the time in milliseconds, at which 63% of the change in voltage (according to highest deflection) is reached. For this, an exponential fit of the course of the membrane voltage from stimulus onset to highest deflection was used (provided R2 > 0.9). The sag index is calculated as follows: change of membrane potential reached at the highest deflection minus change at steady state, divided by the former. The rheobase is defined as the value of a 1 s-long current step as the minimal stimulation sufficient to evoke a single AP or burst in case of burst spiking cells. The AP threshold is defined as the membrane potential at which the slope of the upstroke AP reaches 10 % of the maximum slope of the rising phase. The AP width was determined by the time passed from the half-amplitude and back to the same membrane potential during repolarization. The AHP amplitude was calculated by the difference between threshold and the peak of the hyperpolarization after the AP. The firing pattern of a neuron was assessed by an increasing stimulation of 1 s-long current pulses with an increment of 10 pA until the cell appeared to be saturated by either no further increase in spiking or prominent fluctuations of AP amplitudes. The 10:01 adaptation ratio was calculated as 1 minus the ratio of the average AP counts of the first tenth of the spike train and the last tenth. The introduction of the first term is necessary to accommodate some cases of BS cells, which cease to fire APs in the late segment of the stimulation.

Input resistance and time constant of 23 SST cells recorded with cesium-based solution and clearly identified morphological (m) type (16 Martinotti cells, MC, and 7 non-Martinotti cells, nMC) were used to develop a classifier using MATLAB (MathWorks, Natick, Massachusetts, USA). The model trained was a k-nearest neighbor classifier (3 nearest neighbours, distance measure = Euclidean), which was cross-validated with the leave-one-out method. The validation performance was 82.6 %.

#### Detection and quantification of optically evoked responses

Response latencies were quantified as time difference between stimulus onset and the time point in which the membrane voltage passes 3 times the standard deviation of baseline fluctuations at the pre-stimulus membrane potential. The end of the responses was determined by the return of the membrane voltage back to threshold level. The amplitude was calculated as the maximum deviation of the membrane voltage within the first 50 ms of stimulus onset. The integral was determined by the area delineated by course of the membrane voltage during the response and the baseline resting potential. The time to peak or rising time is calculated by the time difference between crossing of the threshold and time point of the amplitude.

#### Exclusion criteria and cell numbers

Specificity of injections was evaluated by vertical plot profile of YFP-ChR2 signal along the cortical column (see Figure S1). For this purpose, sum projections of confocal image stacks were used. Recordings of cells were excluded if either (i) profiles contained noticeable peaks in laminae which were not supposed to have fiber terminations (such as LIV in POm-injected animals or LI or LVa in VPM injections, 10 cells from 9 animals excluded) or (ii) electrophysiological and anatomical characterization strongly suggested that the recorded cell was not an intended target despite expression of the fluorescent reporter protein (such as an excitatory cell, 2 cells excluded). Analysis of thalamocortical responses included 205 cells from 91 animals.

#### Logistic regression model of response incidence

To better understand the relationship between cell type (VIP vs. SST), home layer (LI, LII/III, LIV, LVA, LVB, LVI) and recording solution (K- vs. Cs-based), we developed a logistic regression model with multinominal predictors for each type of thalamic projection in a stepwise manner using the stepwiseglm function in MATLAB. A significant better fit could be obtained for VPM incidence using the variables intracellular solution and layer location (Chi2 vs. constant: 25.5, *p* < 0.001, McFadden’s R2 0.549).

#### Quantitative comparisons

Two-group comparisons were done in SigmaPlot 13 (Systat Software, San Jose, USA). If not specifically stated otherwise the significance test used was a Mann-Whitney Rank Sum Test. If the distribution of the respective parameter passed a normality test (Shapiro-Wilk), a t-test was used for two-group comparisons. In these cases, the center of the distribution was indicated by the mean and not median. For investigating the effect of SST m-type (MC vs nMC), thalamic nucleus (VPM vs POm) and their possible interaction on synaptic properties, a 2-way-ANOVA was used. Statistical significance with a P-value of 0.05 or smaller in any hypothesis test is indicated by a singular asterisk. All statistical tests, including sample size and degrees of freedom if applicable, are listed in supplemental information (Table S1).
