# Supplementary material for: Functional thalamocortical innervation of VIP- and SST-expressing GABAergic interneurons in mouse barrel cortex
Source: iScience. 2025 Apr 28;28(6):112539. doi: 10.1016/j.isci.2025.112539 (PMC12225920; doi:10.1016/j.isci.2025.112539)
Supplement: Document S1. Figure S1–S5 and Table S1 [file mmc1.pdf]

iScience, Volume *28*

Supplemental information

Functional thalamocortical innervation of VIP- and SST-expressing GABAergic interneurons in mouse barrel cortex

Michael Feyerabend, Mirko Witte, Martin Möck, and Jochen F. Staiger

Supplemental table S1: Statistical figures information

Figure	Groups/Factors	Test	P-value	test value	df	n
2B. 2D	intracellular solution to predict presence of VPM input	regressions with multinominal variables	P = <0.001	statistic vs. constant model:	101	109
2F, 2H	using layer, cell type and intracellular solution to predict presence of POm input	step-wise logistic regressions with multinominal variables	P = 0.097	Chi^2-statistic vs. constant model: 12.1244	87	95
3C	light intensity of evoked VPM responses recorded with K+ based IC solution (SST vs VIP)	Mann-Whitney Rank Sum Test	P = 0.113	U = 201.5	not applicable	23 vs 24
3C	latency of evoked VPM responses recorded with K+ based IC solution (SST vs VIP)	Mann-Whitney Rank Sum Test	P = 0.823	U = 256	not applicable	23 vs 24
3C	integral of evoked VPM responses recorded with K+ based IC solution (SST vs VIP)	Mann-Whitney Rank Sum Test	P = <0.001	U = 110	not applicable	23 vs 24
3C	amplitude of evoked VPM responses recorded with K+ based IC solution (SST vs VIP)	Mann-Whitney Rank Sum Test	P = <0.001	U = 119	not applicable	23 vs 24
3C	time to peak of evoked VPM responses recorded with K+ based IC solution (SST vs VIP)	Mann-Whitney Rank Sum Test	P = 0.221	U = 218	not applicable	23 vs 24
3E	light intensity of evoked POm responses recorded with Cs+ based IC solution (SST vs VIP)	Mann-Whitney Rank Sum Test	P = 0.011	U = 255.5	not applicable	27 vs. 32
3E	latency of evoked POm responses recorded with Cs+ based IC solution (SST vs VIP)	Mann-Whitney Rank Sum Test	P = 0.030	U = 289	not applicable	27 vs. 32
3F	integral of evoked POm responses recorded with Cs+ based IC solution (SST vs VIP)	Mann-Whitney Rank Sum Test	P = 0.353	U = 371	not applicable	27 vs. 32
3F	amplitude of evoked POm responses recorded with Cs+ based IC solution (SST vs VIP)	Mann-Whitney Rank Sum Test	P = 0.003	U = 235	not applicable	27 vs. 32
3F	time to peak of evoked POm responses recorded with Cs+ based IC solution (SST vs VIP)	Mann-Whitney Rank Sum Test	P = 0.014	U = 271	not applicable	27 vs. 32

4C	latency of evoked SST responses recorded with Cs+ based IC solution (VPM vs POm)	Mann-Whitney Rank Sum Test	P = 0.001	U = 147	not applicable	24 vs. 27
4D	integral of evoked SST responses recorded with Cs+ based IC solution (VPM vs POm)	Mann-Whitney Rank Sum Test	P = 0.12	U = 232	not applicable	24 vs. 27
4D	amplitude of evoked SST responses recorded with Cs+ based IC solution (VPM vs POm)	t-test	P = 0.018	t=2.441	49	24 vs. 27
4D	time to peak of evoked SST responses recorded with Cs+ based IC solution (VPM vs POm)	Mann-Whitney Rank Sum Test	P = 0.534	U = 280	not applicable	24 vs. 27
5B	input resistance of SST m-types recorded with recorded with Cs+ based IC solution (MC vs nMC)	Mann-Whitney Rank Sum Test	P = <0.001	U = 243	not applicable	16 vs 7
5B	time constant of SST m-types recorded with Cs+ based IC solution (MC vs nMC)	Mann-Whitney Rank Sum Test	P = 0.002	U = 240	not applicable	16 vs 7
5D	time to peak of evoked responeses recorded with Cs+ based IC solution by predicted SST m-types (MC vs nMC)	2X2 anova with interaction	P (Thal. P.) = 0.4327, P (M-Type) = 0.0356, P(inter.) = 0.1948	F(Thal. P.) = 0.63, F(M-Type) = 4.69, F(inter.) = 1.73	(1, 46)	50 (24 VPM vs. 26 Pom, pMC = 34, pNMC = 16)
5E	amplitude of evoked responses recorded with Cs+ based IC solution by predicted SST m-types and thalamic projection (MC vs nMC; VPM vs POm)	2X2 anova with interaction	P (Thal. P.) = 0.0029, P (M-Type) = 0.4901, P(inter.) = 0.0425	F(Thal. P.) = 9.87, F(M-Type) = 0.48, F(inter.) = 4.35	(1, 46)	50 (24 VPM vs. 26 Pom, pMC = 34, pNMC = 16)
SF 4E	input resistance by SST e-type recorded with K+ based IC solution (Class 1 vs Class 2)	Mann-Whitney Rank Sum Test	P = <0.001	U = 47	not applicable	15 vs 25
SF 4E	tau by SST e-type recorded with K+ based IC solution (Class 1 vs Class 2)	Mann-Whitney Rank Sum Test	P = <0.001	U = 18	not applicable	15 vs 25
SF 4E	rheobase by SST e-type recorded with K+ based IC solution (Class 1 vs Class 2)	Mann-Whitney Rank Sum Test	P = <0.001	U = 44	not applicable	15 vs 25
SF 4E	action potential width by SST e-type recorded with K+ based IC solution (Class 1 vs Class 2)	Mann-Whitney Rank Sum Test	P = <0.001	U = 37	not applicable	15 vs 25

SF 4E	fAHP amplitude by SST e-type recorded with K+ based IC solution (Class 1 vs Class 2)	Mann-Whitney Rank Sum Test	P = 0.003	U = 66	not applicable	15 vs 25

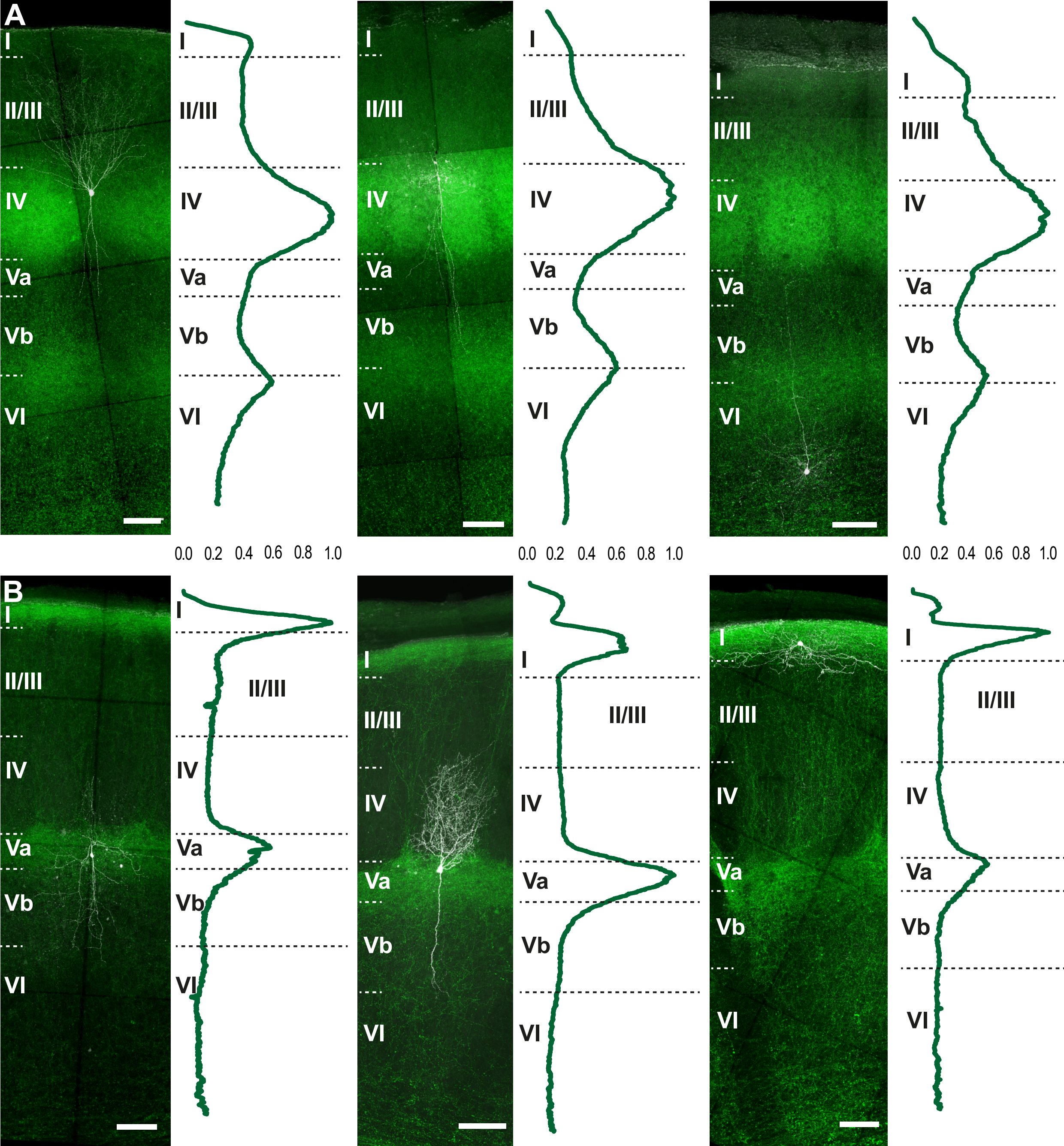

A
I
II/III
IV
Va
Vb
VI
0.0 0.2 0.4 0.6 0.8 1.0
B

Suppl.-Figure 1: Evaluating specificity of stereotaxic injections via laminar thalamocortical projection pattern profiling

A and **B** show maximum intensity projections of representative examples of patched cells in grayscale and the underlying pattern of transduced TCAs revealed by YFP expression in green, for VPM and POm, respectively. Images are accompanied by the normalized vertical plot profile, obtained from sum z-projections of fluorescence intensity. These plots were used to evaluate the specificity of the injection (see text). *Scale bar*: 100 µm.

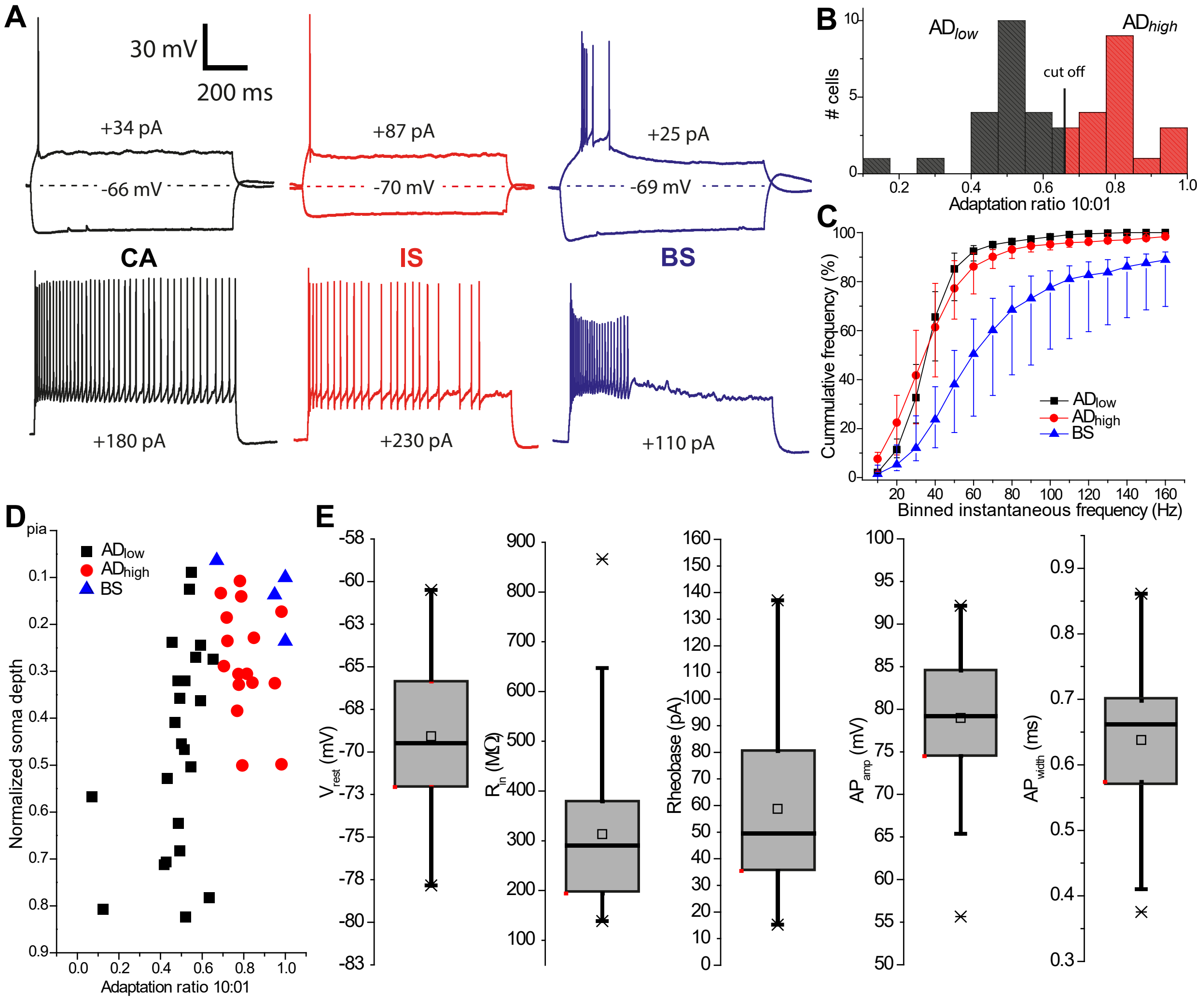

A
30 mV
200 ms
+34 pA
-66 mV
+87 pA
-70 mV
+25 pA
-69 mV
CA
IS
BS
+180 pA
+230 pA
+110 pA
B
ADlow
ADhigh
cut off
cells
Adaptation ratio 10:01
C
Cummulative frequency (%)
Binned instantaneous frequency (Hz)
ADlow
ADhigh
BS
D
pia
Normalized soma depth
Adaptation ratio 10:01
ADlow
ADhigh
BS
E
Vrest (mV)
Rin (MΩ)
Rheobase (pA)
APamp (mV)
APwidth (ms)

Suppl.-Figure 2: Intrinsic properties of VIP cells recorded with K^+-based intracellular solution

A Examples of cells with archetypical firing pattern associated with VIP cells. Hyperpolarizing current step in all cells is −100 pA. **B** Histogram of spike adaptation ratio of non-BS VIP cells. The two peaks in the distribution suggests that there are two populations with different adaptation behavior. This metric was used as objective criterium to distinguish between f continuous adapting (CA) cells (which showed lower values), whereas irregular spiking cells (IS) showed stronger adaptation due to longer stretches of ISIs. **C** Dynamic frequency range of the three different firing pattern phenotypes. Plot indicates the median of the cumulative fraction of the total instantaneous frequency. Data is resampled into 10 Hz bins. Error bars mark the interquartile range (IQR). **D** Scatter plot showing the relationship between adaptation ratio and normalized depth of the soma. BS and IS cells are concentrated in the upper half of the cortex. **E** Distributions of selected intrinsic electrophysiological properties irrespective of firing pattern. Averages are indicated by small hollow squares (n = 48).

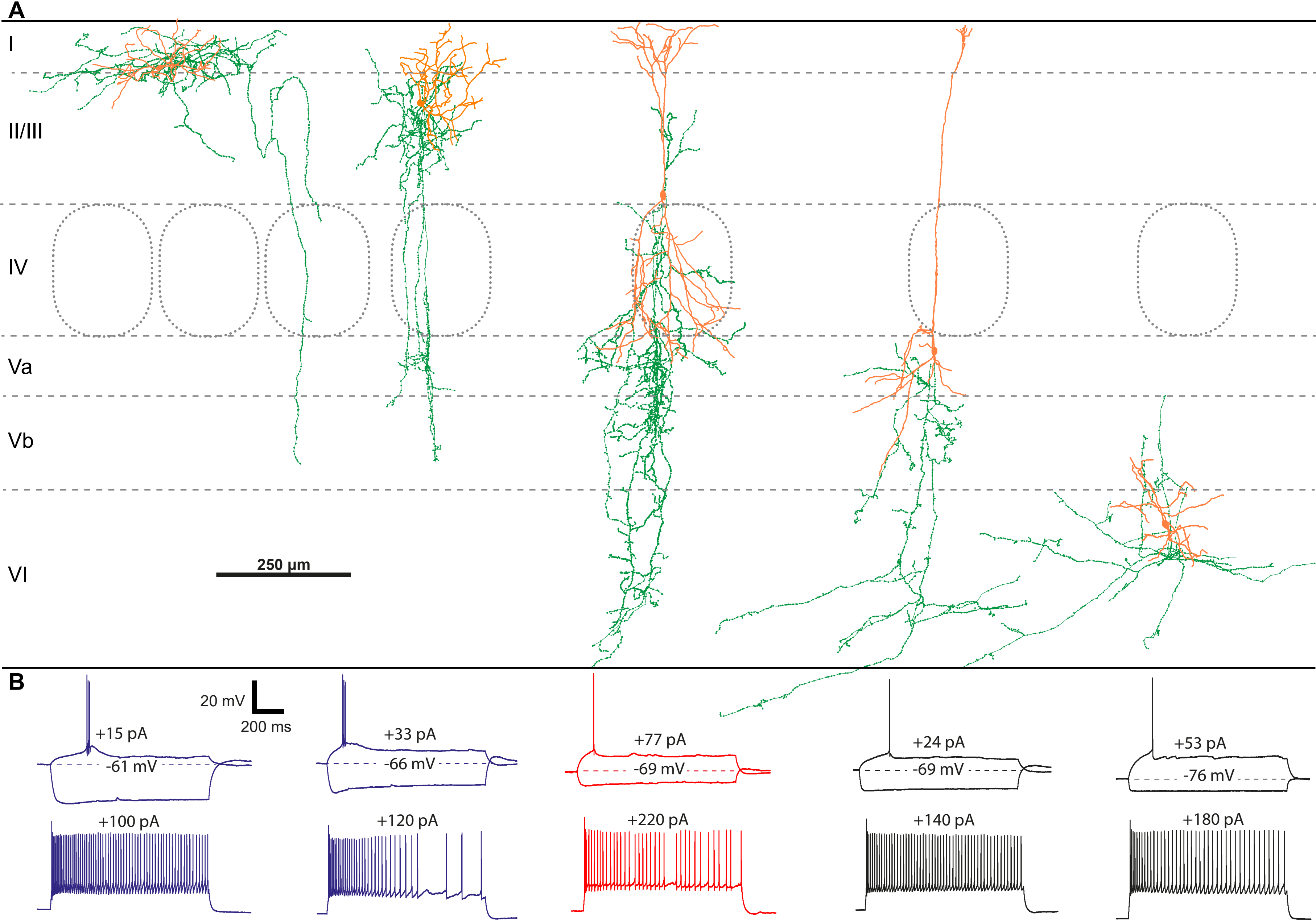
A
I
II/III
IV
Va
Vb
VI
250 µm
B
20 mV
200 ms
+15 pA
-61 mV
+100 pA
+33 pA
-66 mV
+120 pA
+77 pA
-69 mV
+220 pA
+24 pA
-69 mV
+140 pA
+53 pA
-76 mV
+180 pA

Suppl.-Figure 3: Examples of VIP cell morphology and corresponding intrinsic electrophysiological properties.

A Reconstructed cells from various layers. Cells are characterized by a descending axon, usually ramifying locally and then extending into layers below, towards the white matter. From deep LII/III until LVa (see cell 3 and 4) somatodendritic configurations are a blend of bipolar and bitufted and show prominent vertically extended dendritic arbors. The more the soma is located at the poles of the column, the smaller the dendritic spanning field (see cell 1 and 5). With the exception of deep infragranular cells, VIP cells have at least one dendrite targeting LI, where it shows considerable branching. **B** Corresponding firing patterns of cells, introduced in A, in the same color code used in Suppl.-Fig. 2; Hyperpolarizing current step in all cells is −100 pA. Note that cells showing the BS and IS firing pattern are found in the upper half of the cortical column.

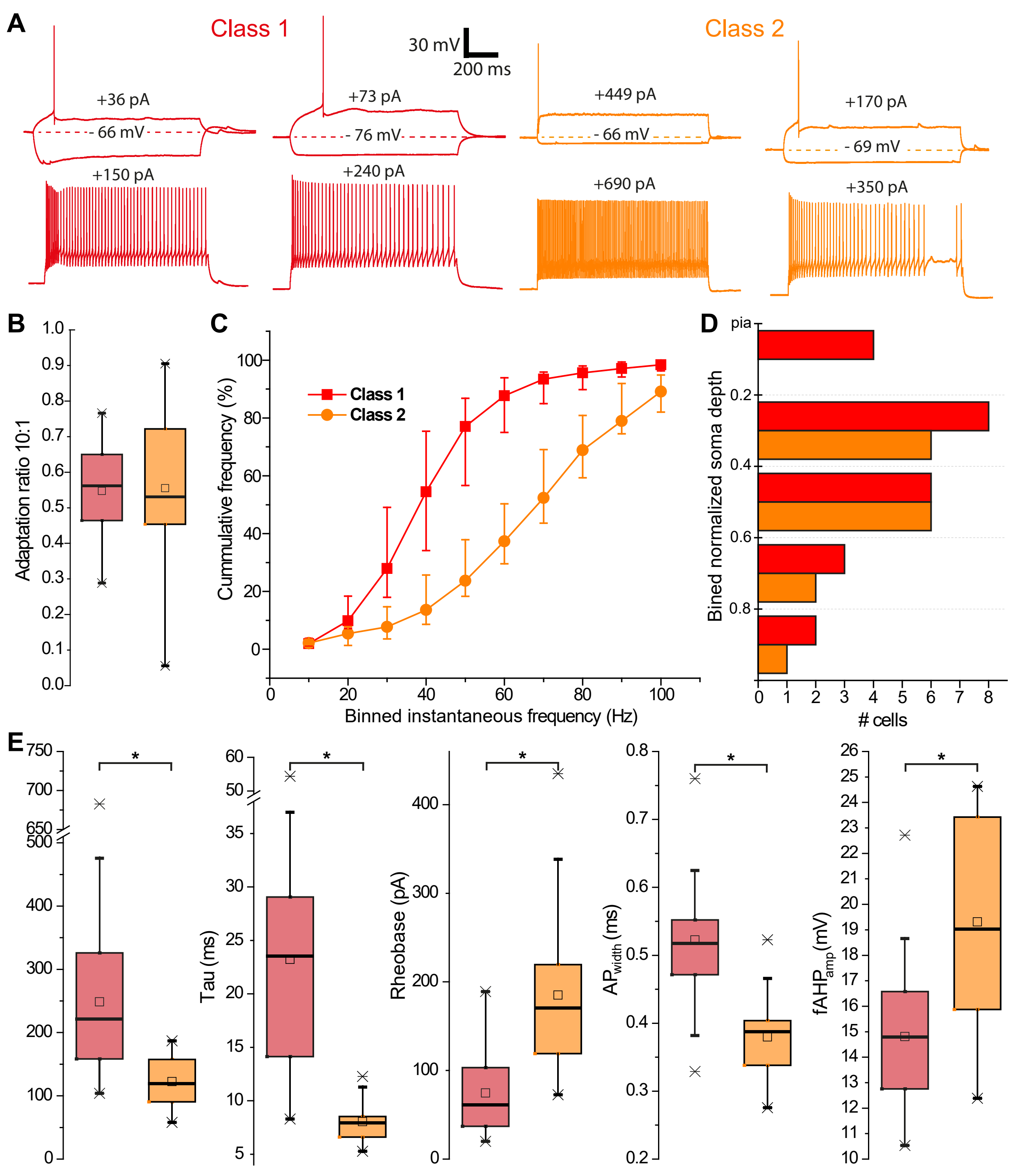
A
Class 1
Class 2
30 mV
200 ms
+36 pA
- 66 mV
+150 pA
+73 pA
- 76 mV
+240 pA
+449 pA
- 66 mV
+690 pA
+170 pA
- 69 mV
+350 pA
B
Adaptation ratio 10:1
C
Class 1
Class 2
Cummulative frequency (%)
Binned instantaneous frequency (Hz)
D
pia
Bined normalized soma depth
cells
E
*
Tau (ms)
Rheobase (pA)
AP_{width} (ms)
$fAHP_{amp}$ (mV)

Suppl.-Figure 4: SST cell firing patterns recorded with K^+-based intracellular solution can by divided into two distinct classes.

A Representative examples of different firing patterns of SST cells: first two cells in red are in Class 1, one being a LTS cell, the second a CA. The other two cells in orange are associated with Class 2. The first one shows the stereotypical FS phenotype, whereas the other is a stuttering quasi-FS. **B** Firing pattern adaptation of both classes is on average similar, but Class 2 shows a higher variance. **C** Dynamic frequency range of the two classes visualized by their cumulative instantaneous frequency. Symbols indicate the median the error bars mark the IQR. **D** Distribution of cells according to normalized distance to pia and subclass: Class 1 is more prevalent in the upper half of supragranular layers. Class 2 is most frequent around the granular layer. **E** Intrinsic subthreshold properties and spike waveform between SST classes are significantly different: R_{in}, Tau, Rheobase, AP_{width}, all $P < 0.001$, absolute amplitude of fAHP, $P = 0.003$. Class 1 $n = 15$, Class 2 $n = 25$.

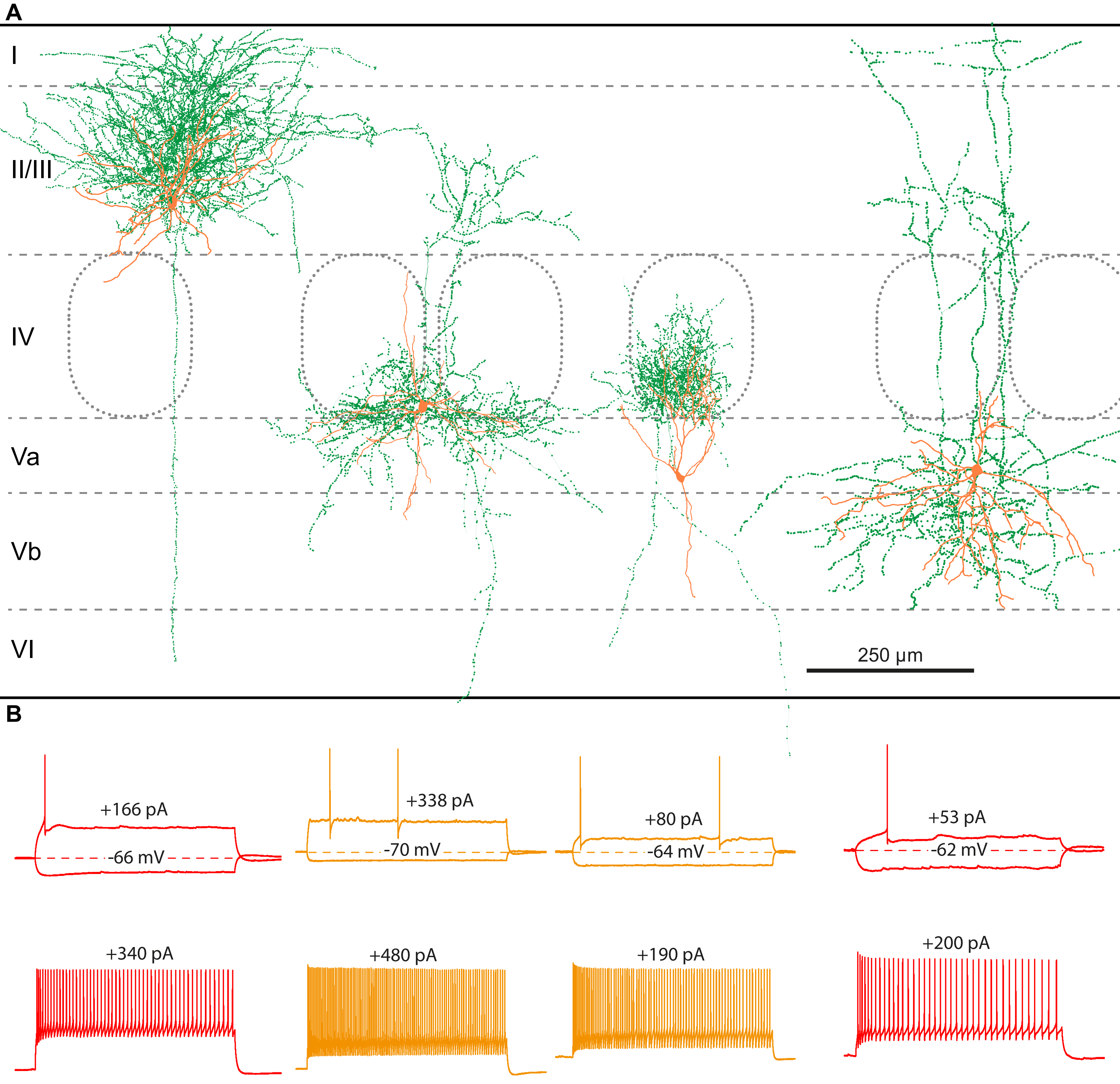
A
I
II/III
IV
Va
Vb
VI
250 μm
B
+166 pA
-66 mV
+338 pA
-70 mV
+80 pA
-64 mV
+53 pA
-62 mV
+340 pA
+480 pA
+190 pA
+200 pA

Suppl.-Figure 5: Examples of SST cell morphology and corresponding intrinsic properties.
A Four examples of SST cell morphology: generally, SST cells are characterized by a multipolar somatodendritic configuration and high axonal density. The first and last cell are MCs characterized by substantial axon branching in L1. The axon of the L5 MC is cut several times, hence its projections in L1 are incomplete. The third cell from the left has the X94 morphology (see Ma et al., 2006; Naka et al., 2019), whereas the second has features reminiscent of a basket cell. **B** Corresponding firing patterns of cells, introduced in A, in the same color code for subclass used in 3.6; Hyperpolarizing current step in all cells is −100 pA. Morphology and firing pattern show a high correspondence: MCs are overwhelmingly associated with Class I, whereas X94 and other non-MC cells are exclusively Class II.
